# Unicentric Castleman Disease Presenting with a Ruptured Pseudoaneurysm within the Tumor: A Case Report

**DOI:** 10.70352/scrj.cr.25-0497

**Published:** 2026-01-30

**Authors:** Toshiki Matsui, Kohei Kawagita, Kazuki Nomura, Maki Hamaguchi, Natsuki Hashiba, Naoya Tsuji, Hirotaka Shibuya, Yosuke Yamauchi, Daisuke Noguchi, Keita Sato, Yoshihisa Tamura, Ryosuke Desaki, Koji Kumamoto, Koji Fujii, Koji Takahashi, Tsukasa Kusuta, Toji Murabayashi, Shinya Sugimoto, Junji Uraki, Mari Ueda, Tadashi Yabana

**Affiliations:** 1 Department of Surgery, Ise Red Cross Hospital, Ise, Mie, Japan; 2 Department of Gastroenterology, Ise Red Cross Hospital, Ise, Mie, Japan; 3 Department of Radiology, Ise Red Cross Hospital, Ise, Mie, Japan; 4 Department of Pathology, Ise Red Cross Hospital, Ise, Mie, Japan

**Keywords:** Castleman disease, ruptured pseudoaneurysm, hypervascular tumor, transcatheter arterial embolization, retroperitoneum

## Abstract

**INTRODUCTION:**

Castleman disease is a lymphoproliferative disorder of unknown etiology, typically reported as a hypervascular tumor. Here, we report the first known case of unicentric Castleman disease in which a pseudoaneurysm developed within the tumor and ruptured spontaneously.

**CASE PRESENTATION:**

A 55-year-old male was hospitalized due to the sudden onset of epigastric pain. Contrast-enhanced CT revealed a 60-mm hypovascular mass located on the dorsal side of the pancreas, with evidence of extravasation within the tumor. Emergency angiography was performed; nonetheless, the pseudoaneurysm was not clearly identified. The patient was administered antihypertensive therapy and discharged. He was subsequently referred to our hospital for further examination and treatment. Upon reviewing the angiographic images obtained at the previous hospital, a pseudoaneurysm was retrospectively identified in a small branch of the splenic artery. Follow-up contrast-enhanced CT at our hospital showed slight tumor shrinkage and resolution of the extravasation. Although the ventral region of the tumor showed marked enhancement, no noticeable enhancement was observed in the dorsal region. Based on imaging findings, a pancreatic neuroendocrine tumor was suspected. While endoscopic ultrasonography fine-needle tissue acquisition was performed twice, a definitive diagnosis could not be made. Suspecting a pancreatic neuroendocrine tumor, we recommended surgery. The patient underwent anterior radical antegrade modular pancreatosplenectomy. Histopathological findings showed that the tumor had no continuity with the pancreatic tissue and was composed of 2 lesions. The ventral portion of the tumor showed lymphoid tissue proliferation with follicular hyperplasia. Blood vessels traversed the lymphoid follicles, and blood vessel walls showed hyalinization and thickening. No atypical lymphocytes were observed. The dorsal portion of the tumor was necrotic. Based on these findings, the patient was diagnosed with unicentric Castleman disease (hyaline vascular type). At the time of writing, there was no recurrence of the disease 24 months after surgery.

**CONCLUSIONS:**

In Castleman disease, as demonstrated in this case, pseudoaneurysm formation may occur. In cases presenting with an intratumoral pseudoaneurysm, Castleman disease should be considered in the differential diagnosis, and angiography should be included for further evaluation and preoperative treatment.

## Abbreviations

CD
: Castleman disease
EUS-TA
: endoscopic ultrasonography tissue acquisition
HHV
: human herpesvirus
HIV
: human immunodeficiency virus
HV
: hyaline vascular
IL-6
: interleukin-6
MCD
: multicentric Castleman disease
PC
: plasma cell
POEMS
: polyneuropathy, organomegaly, endocrinopathy, M-protein, and skin changes
TAE
: transcatheter arterial embolization
UCD
: unicentric Castleman disease
VEGF
: vascular endothelial growth factor

## INTRODUCTION

CD is a lymphoproliferative disorder of unknown etiology, first reported in 1954 as a solitary thymic lesion in the mediastinal lymph node.^1)^ CD is classified as UCD when the lesion is localized and MCD when the lesion spreads across multiple regions.^2)^ Treatment approaches for CD are classified according to the lesion distribution: surgical resection for UCD, whereas chemotherapy is employed for MCD. Several reports have documented massive bleeding during the surgical resection of UCD,^3,4)^ and preoperative TAE has been reported to be useful in managing this risk.^5–7)^ Elevated levels of inflammatory cytokines, such as IL-6 and VEGF, have been observed in CD^8–11)^ and are thought to contribute to the tumor’s hypervascular nature. Herein, we report the first case of UCD complicated by the development and spontaneous rupture of a pseudoaneurysm within the tumor, occurring without any trigger prior to surgery.

## CASE PRESENTATION

**Fig. 1:**
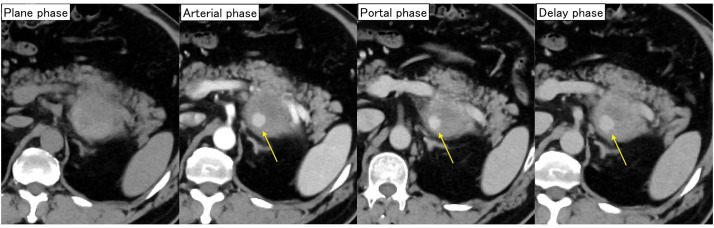
CT scan findings in the previous hospital. A dynamic-enhanced CT scan revealed a 60-mm hypovascular mass on the dorsal side of the pancreas and extravasation inside the mass (arrows).

A 55-year-old male was transferred to another hospital due to the sudden onset of epigastric pain. His medical history included dilated cardiomyopathy, hypertension, and sleep apnea syndrome, and he had no notable family history. He had no history of abdominal trauma prior to this incident. Contrast-enhanced CT scan revealed a 62 × 48-mm hypovascular mass located on the dorsal side of the pancreas with evidence of extravasation (**Fig. 1**). Suspecting rupture of a pseudoaneurysm, an emergency angiogram was performed; however, the pseudoaneurysm was not clearly visualized. He underwent a follow-up CT scan 2 days after the aneurysm rupture, which confirmed that the extravasation had resolved, and no additional endovascular treatment was performed. Consequently, he was treated with antihypertensive therapy and discharged from that hospital. Two months later, he was referred to our hospital for further examination and treatment.

**Fig. 2:**
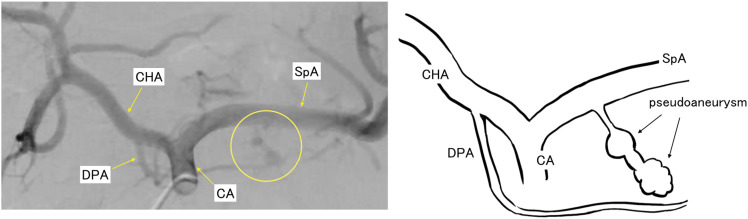
Angiographic findings (in the previous hospital) and their schemas. Rupture of a pseudoaneurysm in the dorsal pancreatic artery was confirmed retrospectively (circle). CA, celiac artery; CHA, common hepatic artery; DPA, dorsal pancreatic artery; SpA, splenic artery

**Fig. 3:**
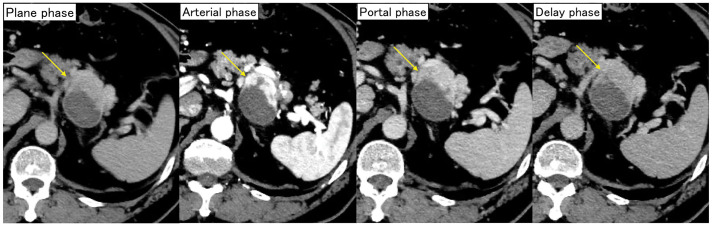
Dynamic-enhanced CT scan findings 2 months after the onset of abdominal pain. A CT scan showed a 55 × 42-mm mass on the dorsal side of the pancreas, and a marked contrast enhancement was observed in the ventral area of the tumor (arrows), but no noticeable contrast enhancement was observed in the dorsal area of the tumor.

**Fig. 4:**
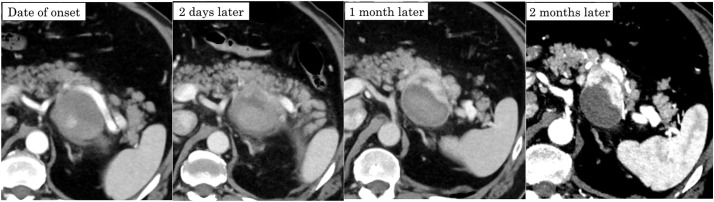
Changes in CT scan findings of the lesion over time. All images were from the arterial phase. Compared with later CT scans, the scans on the day of onset and 2 days later showed weaker contrast enhancement in the ventral area of the tumor, which was presumed to be due to vascular spasm.

**Fig. 5:**
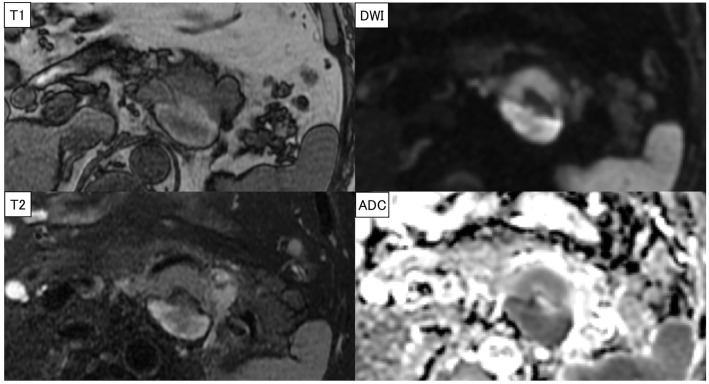
MRI findings. The ventral side of the tumor showed a low signal on T1-weighted images, mildly high signal on T2-weighted images, and high signal on DWIs, suggesting a neuroendocrine or serous cystic tumor. The dorsal part of the tumor suggested a hematoma or necrosis. ADC, apparent diffusion coefficient; DWI, diffusion-weighted image

**Fig. 6:**
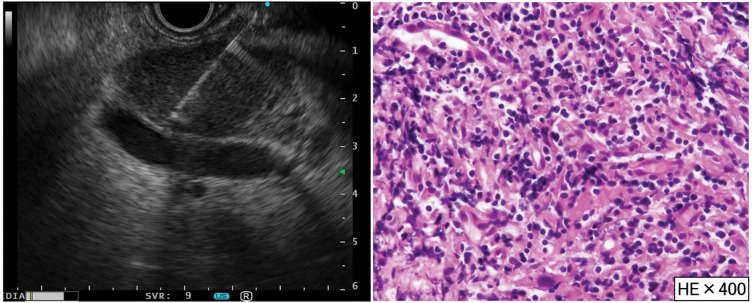
EUS-FNA and pathological findings. EUS-FNA was performed twice for each tumor. Pathological findings showed the infiltration of small lymphocytic cells; however, no tumor cells were observed in the specimen. EUS-FNA, endoscopic ultrasonography fine-needle aspiration; HE, hematoxylin and eosin

Upon admission to our hospital, a retrospective review of the previous angiogram confirmed the presence of a pseudoaneurysm in a small branch of the splenic artery (**Fig. 2**). Contrast-enhanced CT at our hospital showed a 55 × 42-mm mass on the dorsal side of the pancreas. The arterial phase demonstrated marked enhancement in the ventral area of the tumor, whereas no noticeable contrast enhancement was observed in the dorsal area (**Fig. 3**). Because there were considerable differences between the initial CT scans and the follow-up CT scans at our hospital, all follow-up CT scans were arranged in chronological order (**Fig. 4**). Laboratory tests showed no elevation of tumor markers. MRI revealed high signal intensity on T2-weighted images, low signal intensity on T1-weighted images, and reduced diffusion on diffusion-weighted images in the ventral area of the tumor, correlating with the area of marked enhancement on CT (**Fig. 5**). EUS showed a 44-mm hypoechoic mass with a clear border in the tail of the pancreas. The mass was composed of 2 parts: the ventral part was a uniformly solid area, and the dorsal part was a cyst-like area that did not appear on Sonazoid (GE HealthCare Pharma, Tokyo, Japan) imaging. The pancreas and the mass were in contact but not continuous. Based on these imaging findings, a pancreatic neuroendocrine tumor or malignant lymphoma was suspected as a differential diagnosis. To obtain a definitive diagnosis, EUS-TA was performed. However, histopathological findings showed that the tissue obtained by EUS-TA was small and consisted only of lymphocytic cells and fibrous tissue. Tumor cells were not identified in the specimen, and a definitive diagnosis could not be made. A second EUS-FNA was performed, yielding similar results (**Fig. 6**). A pancreatic neuroendocrine tumor was suspected based on the imaging findings, and surgical resection of the tumor was recommended. The patient underwent anterior radical antegrade modular pancreatosplenectomy. After surgery, an International Study Group on Pancreatic Fistula (ISGPF) grade B pancreatic fistula developed; however, the complication was cured with conservative treatment, and the patient was discharged on the 14th POD.

**Fig. 7:**
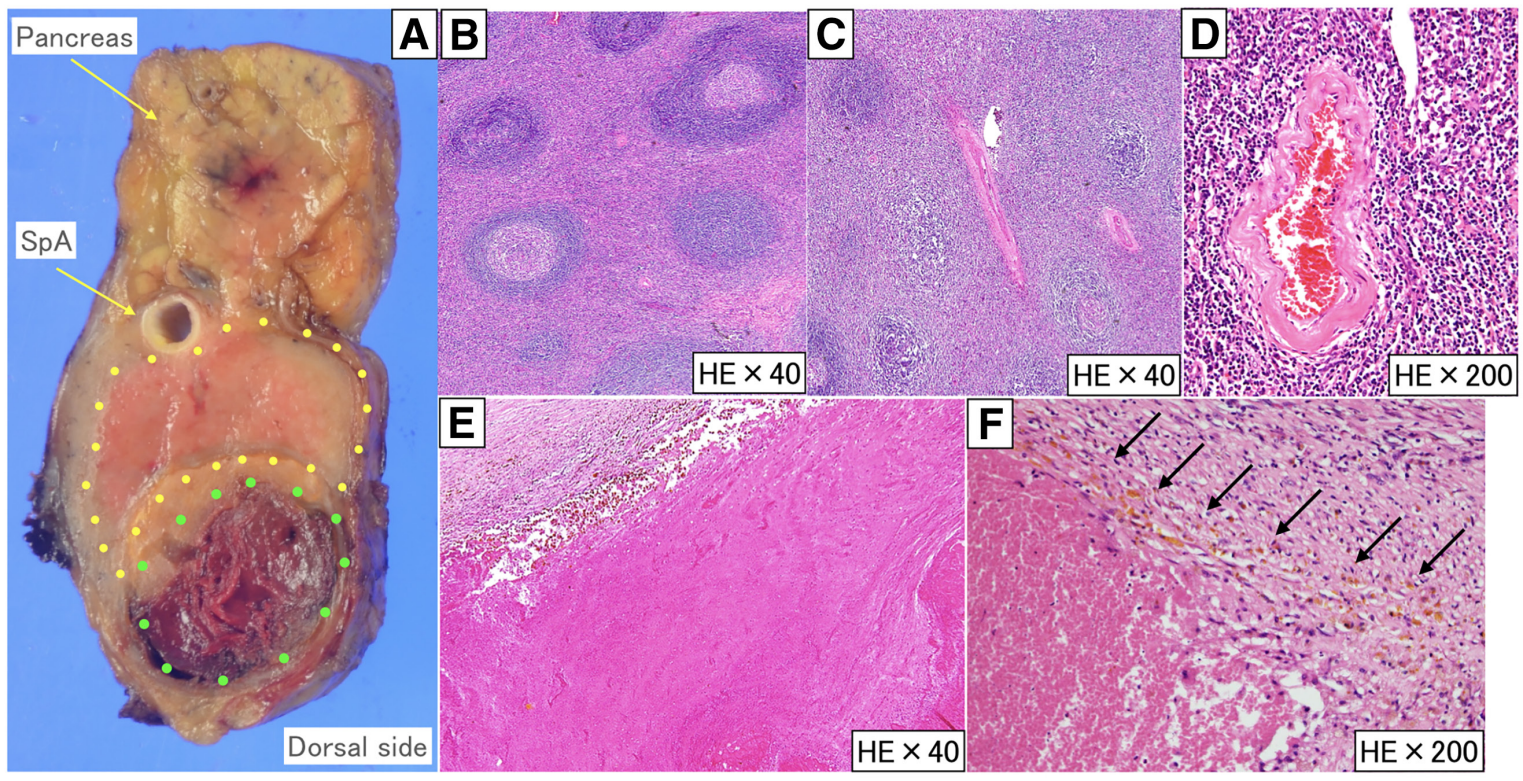
Pathological findings. The yellow dotted area is a lesion of Castleman disease, and the green dotted area is necrotic tissue (**A**). In the yellow dotted area, there is a proliferation of lymphoid tissue accompanied by large and small follicular hyperplasia, as well as development of the mantle layer, atrophy of the germinal center (**B**), and blood vessels running between the lymph follicles (**C**). The blood vessel wall showed hyalinization and thickening (**D**). The green dotted area was necrotic tissue (**E**). The brown area surrounding the necrotic tissue (arrow) represents hemosiderin deposition, supporting the interpretation that the necrotic region is a sequela of prior bleeding (**F**). HE, hematoxylin and eosin; SpA, splenic artery

Pathological findings showed that the ventral side of the tumor had no continuity with the pancreatic tissue and was composed of 2 lesions (**Fig. 7A**): a yellow-white nodule (yellow dotted area) and a brown nodule (green dotted area). In the yellow-white nodules, the proliferation of lymphoid tissue was accompanied by the hyperplasia of lymphoid follicles. In these lymphoid follicles, the development of the mantle layer and atrophy of the germinal center were observed (**Fig. 7B**). The blood vessels ran between the lymph follicles (**Fig. 7C**), and the blood vessel walls showed hyalinization and thickening (**Fig. 7D**). No proliferation of PCs or atypical lymphocytes was observed. The brown nodule was necrotic tissue, and the hematoma formed by rupture of the pseudoaneurysm had become necrotic (**Fig. 7E** and **7F**). The ruptured aneurysm was not identified in the specimens, which was thought to be due to tissue degeneration associated with the 3-month interval between the pseudoaneurysm rupture and the surgical resection. Based on these findings, the patient was diagnosed with UCD (HV type). At the time of writing, there was no recurrence of the disease 24 months after surgery.

## DISCUSSION

CD is a lymphoproliferative disorder of unknown etiology that was first reported in 1954 as a solitary thymic lesion in the mediastinal lymph nodes.^1)^ MCD is further classified as HHV-8–associated MCD, caused by HHV infection in the setting of immunodeficiency due to HIV infection; POEMS syndrome-associated MCD, characterized by polyneuropathy, organomegaly, endocrinopathy, M-protein, and skin changes (POEMS); and idiopathic MCD, which does not fall under either HHV-8–associated or POEMS syndrome-associated MCD.^12,13)^ Histopathologically, CD can be classified into 3 types: the HV type, characterized by follicular hyperplasia and hyalinized blood vessels penetrating the germinal centers; the PC type, in which PCs proliferate in a sheet-like manner in the interfollicular regions of the lymph nodes; and the mixed type, which is a mixture of both types.^14)^ It has been reported that 90% of UCD cases are of the HV type, while most MCD cases are of the PC type.^15)^ The incidence rates of HV UCD have been reported to be 34.2% in the abdominal cavity, 23.7% in the mediastinum, and 15.8% in the retroperitoneum.^16)^

In CD, mature B cells and PCs are increased in the lymph nodes, especially in the PC and mixed types, and many symptoms are attributed to the excessive production of the inflammatory cytokine IL-6.^8)^ IL-6-mediated inflammation activates inflammatory cells, such as monocytes, macrophages, and T lymphocytes, which secrete VEGF and other inflammatory cytokines involved in the proliferation and survival of endothelial cells, promoting angiogenesis. Nishi and Maruyama reported elevated VEGF levels in CD.^11)^ In experiments using short-term culture systems of lymph nodes from patients with HV- and PC-type UCD, clonal chromosomal abnormalities were observed in stromal cells, including follicular dendritic cells and myoid cells.^17,18)^ These cells may also produce IL-6 and contribute to angiogenesis and disease pathogenesis.^13)^

Our case is novel because a pseudoaneurysm formed in the tumor and spontaneously ruptured without any triggers. In a search of the PubMed and Ichushi (Japanese) databases using the search term “[(Castleman disease) AND (bleeding) OR (hemorrhage)],” to date, although there have been several case reports of massive bleeding in surgery, to the best of our knowledge, there has been no similar case reported. We speculated that the causes of the formation and rupture of the pseudoaneurysm were increased angiogenesis due to the mechanism mentioned above, vulnerability of hyalinized blood vessels, and a medical history of hypertension.

Talat et al. reported a systematic review of 404 published CD cases; 77.0% of the patients with UCD underwent surgical treatment. According to this report, the overall survival rate of patients with UCD is 95.3%. However, the mortality rate of the complete resection group was 3.8%, and that of the incomplete resection group, such as the diagnostic biopsy group, was 17.6%, which was significantly higher than that of the complete resection group. In the multivariate analysis of the prognosis of UCD, only complete surgical resection of the lesion was associated with prognosis.^19)^ Therefore, complete resection of the lesion is important.

There have been several reports of massive bleeding during resection (especially in mediastinal lesions), CD is generally considered a hypervascular tumor.^3,4)^ Several papers have reported that preoperative TAE is useful to reduce intraoperative blood loss.^5–7)^ In our case, angiography was performed at another hospital when the pseudoaneurysm ruptured; however, the pseudoaneurysm could not be identified at that time, and TAE was not performed. If a clear pseudoaneurysm had been confirmed, TAE should have been performed regardless of his condition to prevent rebleeding.

## CONCLUSIONS

Herein, we report the first case of UCD in which a pseudoaneurysm formed in the tissue and ruptured spontaneously. CD is generally known to be a hypervascular tumor that can form pseudoaneurysms, as observed in this case. Therefore, in cases presenting with an intratumoral pseudoaneurysm, CD should be considered in the differential diagnosis, and angiography should be included as part of further evaluation and preoperative treatment.

## DECLARATIONS

### Availability of data and materials

The dataset supporting the conclusions of this article is included within the article.
